# Invasive cells in animals and plants: searching for LECA machineries in later eukaryotic life

**DOI:** 10.1186/1745-6150-8-8

**Published:** 2013-04-04

**Authors:** Katarína Vaškovičová, Viktor Žárský, Daniel Rösel, Margaret Nikolič, Roberto Buccione, Fatima Cvrčková, Jan Brábek

**Affiliations:** 1 Department of Cell Biology, Faculty of Science, Charles University in Prague, Vinicna 7, 128 43, Prague 2, Czech Republic; 2 Department of Experimental Plant Biology, Faculty of Science, Charles University Prague, Vinicna 5, 128 43, Prague 2, Czech Republic; 3 Department of Human and Environmental Sciences, School of Life and Medical Sciences, University of Hertfordshire, College Lane Campus, Hatfield, AL10 9AB, United Kingdom; 4 Tumour Cell Invasion Laboratory, Consorzio Mario Negri Sud, S. Maria Imbaro, Chieti, 66030, Italy

**Keywords:** Invasiveness, Invadopodia, Pollen tube, Neurite, GTPases, Actin, Secretory pathway

## Abstract

Invasive cell growth and migration is usually considered a specifically metazoan phenomenon. However, common features and mechanisms of cytoskeletal rearrangements, membrane trafficking and signalling processes contribute to cellular invasiveness in organisms as diverse as metazoans and plants – two eukaryotic realms genealogically connected only through the last common eukaryotic ancestor (LECA). By comparing current understanding of cell invasiveness in model cell types of both metazoan and plant origin (invadopodia of transformed metazoan cells, neurites, pollen tubes and root hairs), we document that invasive cell behavior in both lineages depends on similar mechanisms. While some superficially analogous processes may have arisen independently by convergent evolution (e.g. secretion of substrate- or tissue-macerating enzymes by both animal and plant cells), at the heart of cell invasion is an evolutionarily conserved machinery of cellular polarization and oriented cell mobilization, involving the actin cytoskeleton and the secretory pathway. Its central components - small GTPases (in particular RHO, but also ARF and Rab), their specialized effectors, actin and associated proteins, the exocyst complex essential for polarized secretion, or components of the phospholipid- and redox- based signalling circuits (inositol-phospholipid kinases/PIP2, NADPH oxidases) are aparently homologous among plants and metazoans, indicating that they were present already in LECA.

**Reviewer:** This article was reviewed by Arcady Mushegian, Valerian Dolja and Purificacion Lopez-Garcia.

## Introduction

Invasive cell growth and migration, including processes such as tumor or immune cell invasion into tissues or neurite outgrowth, is usually considered a topic of biomedically relevant metazoan biology. However, if we understand cellular invasiveness as a general ability to move and/or grow through a heterogenous (semi)solid environment, which may or may not be a living tissue, this phenomenon appears to be widespread among a huge variety of organisms. Recent research, revealed common features and mechanisms of cytoskeletal (mainly actin) rearrangements, membrane trafficking and (not only) GTPase-based signalling, contributing to cellular invasiveness in organisms as diverse as metazoans and plants. Here we summarize such common features shared by organisms connected only via the root of the eukaryotic tree the last common eukaryotic ancestor – LECA [1], aiming towards reconstructing a basic “toolbox” of common, and probably evolutionarily conserved, mechanisms involved in this cellular process.

## Model invasive structures

**Figure 1:**
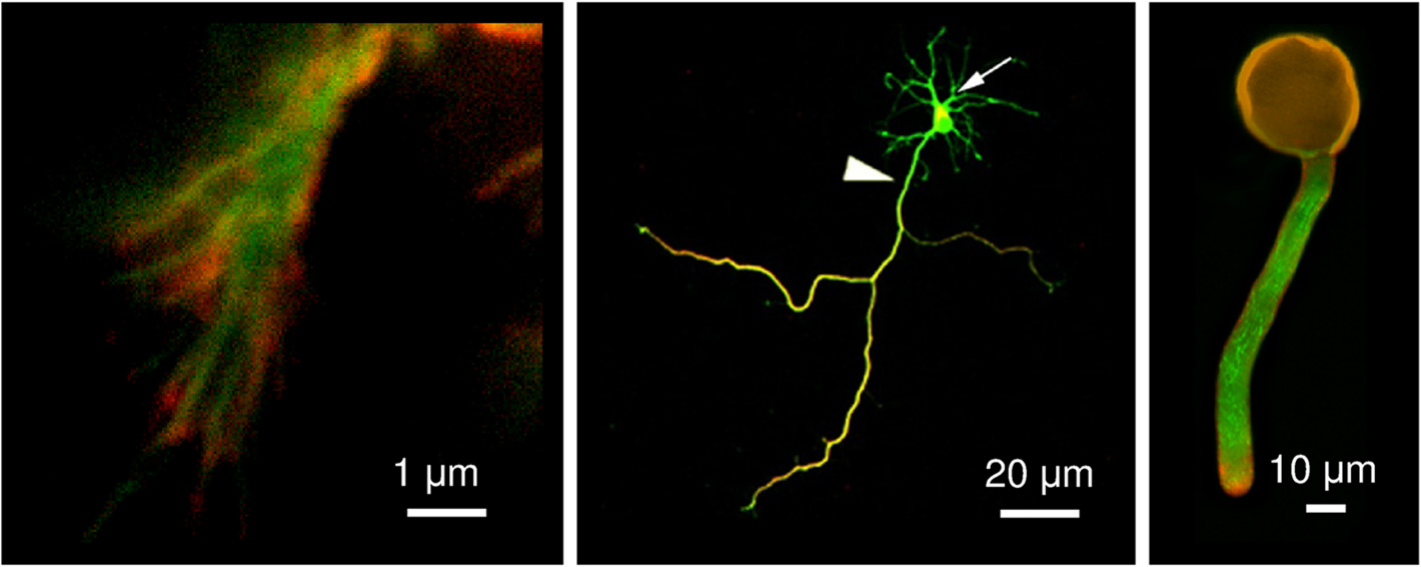
**Model invasive structures. **Left: detailed image of an invadopodial structure from RsK4 sarcoma cells on dermis-based matrix, showing thin F-actin fibers (green) capped with phosphotyrosine signal (red). Middle: a polarised rat embryo hippocampal neuron after three days in culture, expressing cytoplasmic green fluorescent protein (green) and immunolabeled for neuron-specific beta-III-tubulin (red), which marks the axon. Arrow – cell body with dendrites, arrowhead – axon. Right: an *in vitro *cultured tobacco pollen tube labeled with fluorescent antibodies against a component of the Exocyst complex (red) and tubulin (green). The broad-spectrum signal in the pollen grain (located at the top) corresponds to autofluorescence. Tube length can reach several milimeters after a few hours in culture.

While cellular invasiveness is widespread among eukaryotes, we shall focus here only on invasive protrusions generated by model cell types from two major eukaryotic lineages – metazoans and plants. As strange as it might sound, plant cells can namely grow in an invasive manner, as documented in particular for root hairs and pollen tubes. Representative cells of our chosen models are shown in Figure 1.

It has to be stressed that invasive cells are found not only in plants and metazoans, but especially fungi provide a plethora of alternative opisthokont models. The budding yeast *Saccharomyces cerevisiae* has served as a long time paradigmatic cell polarity model that helped to pinpoint the central position of RHO clade GTPases as polarity regulators. Much of the machinery responsible for yeast bud formation is shared also by species capable of true invasive hyphal growth (reviewed e.g. in [2-4]). At least one other eukaryotic supergroup - the chromalveolates - also contains organisms capable of invasive growth, but their characterization is lagging far behind studies in opisthokonts and plants. For instance, penetration of host tissues by *Phytophtora sp*. has been only recently characterized morphologically, and very little is known about the underlying molecular mechanisms [5,6].

Our selection of models is, out of necessity, restricted by the need to maintain this text at a manageable size and within the scope of the authors’area of research, focused on multicellular animals and plants. We therefore chose two metazoan invasive cell types (transformed or tumor cells and neurons) to represent the opisthokonts, and root hairs and pollen tubes as examples of plant cell invasiveness.

### Invadopodia of transformed metazoan cells

Invadopodia (recently reviewed in [7-10] are stable actin-rich protrusions formed at the ventral surface of invasive tumor or transformed cells cultured on appropriate extracellular matrix (ECM) substrates such as gelatine, fibronectin, collagen, or laminin [11] and displaying focalized proteolytic activity towards the substrate [12,13]. Molecular components involved include integrins, elements of signalling machineries, soluble and membrane-bound proteases (including matrix metalloproteases, or MMPs), and prominently actin and actin-associated proteins such as e.g. cortactin [14-18]. Focal degradation of the ECM at invadopodia involves tight integration of the membrane remodelling, trafficking and signalling machineries, similar to initial steps of tumor cell dissemination.

Microscopically invadopodia appear as roundish actin-rich structures at the ventral surface of cells, not confined to the cell periphery, containing cortactin (or dynamin 2, fascin and others) and/or phosphotyrosine, and associated with sites of substrate degradation, [19,20]. Another feature is their extended half-life of up to 2 hours or more [19,21] as compared to podosomes, related protrusive adhesions [22-24]. Recent studies using advanced 3D imaging methods have highlighted the complexity of actin regulation in invadopodia formation and elongation, further elucidated the specific role of some fundamental players (including microtubules in later stages of invadopodia elongation) and provided novel morphological insights [25-27].

We use invadopodia mainly as a model for understanding the roles of the actin remodelling system, the small GTPases, and finally lipids, which are recently entering the picture in big strides.

### Vertebrate pyramidal neurons – a model of normal cell invasion

The main neuronal model of our review will be pyramidal neurons from the mammalian cerebral cortex or hippocampus, which can extend investigative protrusions to facilitate directed migration from their place of origin to their final destination in the forebrain, and subsequently migrate by climbing up scaffolds of specialised progenitor cells – the radial glia [28-31].

Several aspects of neuronal migration resemble forms of locomotion utilised by other metazoan cell types. These basic principles include chemotaxis [32], reorientation of the centrosome and Golgi apparatus to the base of the leading protrusion [33], and coordinated reorganisation of the F-actin and microtubule cytoskeletons enabling directed vesicle trafficking towards the leading edge [34-38]. Regulated attachment and dissociation takes place between migrating neurons and the radial glia on which they move, through integrin-rich adhesions and gap junctions [39,40], followed by terminal dissociation from radial glia through specialised molecules such as Secreted protein acidic and rich in cysteine (SPARC)-like 1 and reelin, allowing cessation of migration once neurons have reached their final destination [41,42]. Perturbation of these processes is associated with misdirected and/or arrested migration of pyramidal neurons [43-45].

Pyramidal neurons isolated from rodent embryos can undergo a sequential process of maturation *in vitro* on adhesive substrates such as laminin or poly-lysine [46-49], developing into mature cells with a single axon and multiple dendrites. This model system has been mainly used for the study of axon specification, though it may have some limitations [50,51]. For instance, the role of centrosome positioning, or distinguishing signals that polarise the cell from those that promote neurite outgrowth remains controversial [45,51-56]. Nevertheless, post-mitotic neurons are one of the best models for studying the coordinated interplay between the extracellular environment and internal signals in normal cell invasiveness.

### Plant cell invasiveness: root hairs and pollen tubes

The two best studied invasive plant cell types are root hairs and pollen tubes, which elongate by tip growth and penetrate rather complex environments. Root hairs explore random micro-spaces between soil particles, while the growing pollen tube tip, guided by chemotaxis, invades highly organized live pistil tissues to deliver sperm cells to their two targets within the female gametophyte. While the chemotropic guidance is reminiscent of metazoan cell invasiveness, the molecules involved, such as pectins and cystein-rich lipid-transfer protein-like peptides [57], are very different, indicating evolutionary convergence rather than conservation. In another case of convergence with invasive metazoan cells, invasion of pollen tubes into intracellular spaces of the transmitting tract involves secretion of extracellular matrix-loosening enzymes [58]. For instance, xylanases released from pollen grains and expansins secreted by the growing tube help to drill a passage through the cell walls of the transmitting tract in maize [59]. Fortunately, both cell types can be grown *in vitro* and studied in the absence of the complex matrix that is being invaded *in situ*.

Plant cells share some features absent in metazoans, in particular the presence of a (semi-)rigid cell wall and a water solution-filled vacuole, which together produce outward pressure at the cytoplasmic membrane-cell wall interface, known as turgor and contributing, together with local changes in mechanical properties of the cell wall to cell expansion or its regulation [60,61]. The plethora of plant-specific cell wall-modifying activities involved is beyond the scope of this review, albeit they can be understood as ultimate effectors of the conserved pathways discussed below.

Development of both root hairs and pollen tubes begins with specification of their outgrowth site (plant cells lack conventional centrosomes, which can contribute to the positioning of invasive projections e.g., in neurons). While pollen tubes emerge from pre-existing pores within the pollen grain exine, root hairs appear at the rootward end of trichoblasts, a subset of rhizodermis cells that are, in the best characterized model *Arabidopsis thaliana*, developmentally determined by a well-characterized transcriptional circuit [62,63] and polarized by auxin gradient-based signalling [64]. Local relaxation of cell wall results in bulge formation followed by actual root hair outgrowth [65-68].

In both cell types, the growing invasive tip exhibits an apical cytoplasmic “clear zone”, devoid of obvious microscopically visible structures but containing numerous secretory vesicles, where surface expansion associated with vigorous membrane turnover takes place. Dynamic fine F-actin arrays participate in this process, while microtubules may contribute to controlling growth direction (see below).

The growth rate of both pollen tubes and root hairs often oscillates, accompanied by periodic changes in extracellular pH, reactive oxygen species (ROS) and cytoplasmic Ca2+ concentrations [69-71].

Root hairs and pollen tubes are not the only invasive plant cell types. Epidermal pavement cells of aboveground organs undergo localized expansion, coordinated among neighbors and resulting in the formation of puzzle-like interlocked lobes. Recent studies in *Arabidopsis*[72,73] revealed that molecular mechanisms working in pavement cells interdigitation are related to those responsible for invasive growth of root hairs and pollen tubes.

Besides established models such as *Arabidopsis*, the moss *Physcomitrella patens* is gaining on importance due to ease of its genetic manipulations. Moss protonemata, branched chains of cells invading soil or growth medium in an almost mycelium-like fashion, can therefore serve as another interesting model system for the study of plant cell invasiveness. However, as the bulk of data on plant cell invasiveness comes from root hairs and pollen tubes, we focus mainly on these two models.

## The great small GTPases

The Ras superfamily of small molecular weight GTPases controls fundamental cellular functions including those essential for invasive growth. Due to very slow spontaneous intrinsic GTP hydrolysis they act as binary molecular switches, converting between an active, guanosine triphosphate (GTP)-bound state, interacting with a number of effector proteins and thus promoting cellular responses, and an inactive, guanosine diphosphate (GDP)-bound state. Transitions between these states are catalyzed by GTPase-activating proteins (GAPs) stimulating “switch off” hydrolysis of GTP to GDP and by GDP/GTP exchange factor (GEFs) inducing “switch on” charging by fresh GTP [74-76].

### Rac/Rho/Rop – the invasion leaders

Small GTPases of the RHO clade, including opisthokont Rho, Rac, and Cdc42 and plant Rop, participate in the control of cell polarity, motility and also invasive growth via their interaction with various effectors, including protein kinases, actin nucleators, secretory pathway regulators and phospholipases [77-79].

RHO GTPases promote cell invasiveness and motility through their ability to control plasma membrane protrusions and the turnover and integrity of adhesions [77]. In fibroblasts, Rac plays a central role in lamellipodia and membrane ruffling, Rho in stress fibre and focal adhesion formation and Cdc42 controls microspike and filopodia formation and is a master regulator of cell polarity [80-82]. Cdc42 appears to be the main RHO GTPase implicated in the formation of invadopodia, with roles spanning from the regulation of actin remodelling to the control of ECM degradation. Dominant-active mutants of Cdc42 or Rac enhanced both diffuse and dot-like(invadopodia-associated) fibronectin degradation [83] while Cdc42 downregulation suppressed invadopodia formation [21]. Cdc42, but not Rho A or Rac, was detected at invadopodia [19]. The interaction of the multi-domain polarity protein IQGAP1 with proteins of the secretion machinery regulating metalloproteinase activity at invadopodia is triggered by active Cdc42 and is essential for matrix degradation [84].

In neurons, Rac1 is essential for neurite outgrowth *in vivo*. An inactive, dominant Rac1 mutant inhibited axonal growth in Drosophila [85]. Progressive deletion of both Drosophila Rac genes (DRac1 and DRac2) as well as the highly related Mtl gene (Mtl), caused a stage-wise simplification of axon branching, defects in axon guidance and inhibition of axon growth [86-88]. Genetic analysis in Drosophila pinpointed the involvement of Rac1 upstream regulators, including Notch and especially the GEFs Trio, Vav and DOCK180, in the formation of neuronal protrusions [89-91]. Forebrain-specific deletion of mouse Rac1 revealed a specific need for Rac1 during axon guidance, rather than the initiation of neurite outgrowth [92,93]. Functional overlap between Rac1, Rac2 and Rac3 in the developing mammalian cerebral cortex is confirmed by conditional, double deletion of Rac1 and Rac3 [94]. Rac1 loss from neuronal progenitors caused reduced elaboration of lamellipodia, impeding axon growth [95]. Rac1 is also essential for the plasma membrane localisation of the actin regulating protein WAVE, suggesting that in migrating cerebellar neurons lamellipodia are controlled by WAVE and the Arp2/3 complex. While deletion of Rac1 caused defects in the migration of interneurons [92] deletion of Cdc42 caused defects in the positioning of cortical progenitors, thus altering the fate of neurons that arise from them [96,97].

In contrast to the separation of functions between Rac and Cdc42 in metazoans, the sole plant RHO GTPase family of Rops regulates both invasive growth and cell fate [98-100]. Locally activated Rops leading the invasion in plant cells are well documented at very early pre-bulge stages of root hair initiation, as well as during later stages of pollen tube and root hair invasive growth [101-104]. Rop GTPases are activated mostly by a type of GEF proteins absent in Opisthokonts – the PRONE-GEFs [105] - whose activity is regulated by interactions with specific transmembrane Ser/Thr receptor kinases (RLKs; [105,106]). This suggests crucial position of RLK signalling in the regulation of localized (invasive) plant cell growth and provides opportunity for achieving spatial and temporal specifity through choice between diverse members of the enormous plant RLK family [107].

During pollen polarization and germination, extracellular peptides regulate a specific RLKs/PRONE-GEF/Rop signalling module [106]. In root hairs other RLKs/PRONE-GEF/Rop modules may be controlled directly by the interaction with the cell wall macromolecules, their fragments or even by local auxin maxima [68,108]. Also in the developing interdigitated lobed epidermal pavement cells Rop GTPases are crucial for reciprocal invasion of lobes into the neighbouring pavement cells, possibly instructed by local auxin maxima [72,73].

While relatively little is known concerning the role of RHO GTPase regulatory proteins in model invasion cells structures, especially the GEFs arise as possible master regulators of downstream signaling from RHO in diverse systems [109] and might be important players in invasion and metastasis [110]. The limited evidence available points to the Cdc42-specific GEF Fgd1 [111] as a regulator of Cdc42 activity in invadopodia formation [112]. In plants, specific localization of Rop activity down-regulating GAPs to sub-apical region of the plasmalemma of tip-growing cells contributes, along with Rop GDP dissociation inhibitor (GDI), to the sharp restriction of active Rops to the expanding membrane domain [113].

### Arfs and rabs – small GTPases involved in invasion

The ADP-ribosylation factor (ARF) class of small GTPases regulates endosomal membrane trafficking, exocytosis, and actin remodelling at the cell surface across eukaryots [114,115]. In mammals ARF6, but not the other members, participates in acquisition of an invasive phenotype downstream of v-Src activation, by promoting traffic-mediated adherens junction disassembly and epithelial cell migration [116]. ARF6 is localized at invadopodia [117,118], and its expression levels correlate with the invasive phenotype [117]. The mechanism of ARF6 action in invadopodia formation and activity, though not completely defined, appears to be dependent on ERK activation [118,119].

In model neuron systems, ARF6, its GEF (ARNO) and GAPs regulate dendritic branching in a pathway downstream of Rac [120-123]. ARF GTPases, their regulators and their interaction with specific membrane lipids also contribute to invasive growth of plant root hairs [124-126]. AGD1, a class I ADP ribosylation factor GTPase-activating protein, was proven to be important for maintaining straight growth in Arabidopsis root hairs, since loss of function mutations in the *AGD1* gene resulted in wavy root hair growth [125].

The Rab proteins comprise a large family of abundant small GTPases that regulate exocytic and endocytic intracellular trafficking by controlling membrane identity, vesicle formation, motility and fusion [127-129]. At least two members of this family, Rab8 [130-132] and Rab27b [132] have been directly implicated in invasive tumor cell migration. The findings are consistent with a function of these Rabs in invadopodia formation or function and underscore the direct relationship between the intracellular trafficking machinery and ECM degradation at invadopodia. In neurons, much of the research on Rabs has focused on their synaptic activity; some of them, in particular Rab8 (reviewed in [133-135]), are also crucial for neuronal morphogenesis, including invasiveness.

Plant Rab8 homologues, as well as Rab11 homologues (representing 26 out of total 56 Arabidopsis Rabs), control directional cell growth via regulation of exocytosis and recycling of PM proteins [129]. Localization and functions of plant Rab11 and Rab8 proteins is related to plant-specific function of the Trans Golgi Network (TGN) acting at the cross-roads of the exocytotic and endocytotic pathways as an early endosome [136]. Specific Rab11 and Rab8 paralogues and their known PIP kinase effectors (see below) participate in pollen tube and root hair growth in Arabidopsis or tobacco and localize, as expected, to the actively growing domains of the cell surface [137-139].

## The cytoskeleton(s)

Since we intend to compare shared features of plant and metazoan cell invasiveness, and plant cells lack conventional intermediate filaments, we shall focus on the two cytoskeletal systems common to all our models – i.e. the actin microfilaments and, to a lesser extent, also the microtubular cytoskeleton, and their associated proteins.

### Actin is important for the invasion process

Mechanisms generating the forces behind membrane remodelling and protrusion at invadopodia still need to be completely defined; however, microfilaments clearly play a central part. Two main hypotheses have been proposed [140], one assuming that constant growth of a branched actin meshwork propels invadopodia into the underlying matrix, similar to lamellipodia protrusion, while the other suggests that the mechanical force required to overcome substrate stiffness is provided by actin bundles originating from the branched network, akin to filopodia formation. Both dendritic and bundled actin networks, assembled at the sites of contact with the ECM and than forming cores for invadopodia formation, may be relevant in various situations. Elongation of invadopodia apparently relies on the same machinery but requires participation of microtubules to extend invadopodia beyond 5 μm. Intermediate filaments may contribute at later stages [26,140].

Microfilaments are also intimately involved in the initiation and extension of neuronal protrusions. Global application of an F-actin depolymerising drug, cytochalasin D, induced multiple axon-like neurites in unpolarised hippocampal neurons *in vitro*, and axonal characteristics were induced in a single unpolarised neurite after focal application of cytochalasin D. This approach has also provided a useful tool for dissecting actin-related signalling pathways. For instance, inhibition of RHO GTPases caused a similar phenotype as cytochalasin D, suggesting their instrumental role during axon development [50,140]. Furthermore, perturbation of neuronal polarisation e.g. by mislocalization of the p21-activated kinase (Pak-1) can be rescued by cytochalasin D, suggesting signalling pathways downstream of small GTPases that may affect F-actin organisation and/or turnover during neuronal polarisation [141].

Actin dynamics is central also for plant cell invasive growth [142,143]. However, its role in walled cells is likely to differ from that in soft-bodied animal cells or amoebae. Plant actin is important mainly for delivery and targeting of secretory vesicles to the growing plasmalemma domain, enabling at the same time the modification of cell wall properties and thus also changes in its mechanical properties allowing directed cell growth driven by the turgor pressure agains the yielding cell wall.

The very growing tips of pollen tubes (and less distinctly also root hairs or moss protonemata) are apparently free of long visible actin filaments. However, subapical F-actin structures (a “F-actin fringe” of very short and highly dynamic F-actin arrays) are important for secretory vesicle formation, delivery, recycling, and in particular spatial targeting [144-146]. Surprisingly, while complete actin depolymerisation by Latrunculin B or Cytochalasin D inhibits tip growth, mild doses of these inhibitors, destroying the fringe but not actin bundles, cause temporary tip swelling, i.e. enhanced surface expansion [147-149]. Stimulation of exocytosis after gentle actin depolymerization was observed also in a variety of metazoan cells [150-153]. Local balance between soluble G-actin, fine F-actin arrays and F-actin bundles may thus be crucial for maintaining cell expansion localized during invasive growth.

### The role of actin nucleators

The “clasical” actin nucleation machinery based on the Arp2/3 complex, forming branched actin arrays, regulated and stabilized by cortactin, and its activator N-WASP [154,155], is fundamental in the formation of invadopodia (as well as lamellipodia). A FRET-based study showed that N-WASP is active at the base of the invadopodial protrusions in a rat mammary carcinoma cell line [156]. In another transformed cell model, actin in actively degrading invadopodia formed dynamic structures with distinct “head” and “tail” sections, both containing Arp2/3 and N-WASP [19], resembling the actin “comets“associated with invading bacteria [157,158].

Arp2/3-mediated actin nucleation participates also in the advancement of axon growth cones in developing neurites [159,160]. Dendritic spines and their precursors in hippocampal neurons contain Arp2/3-nucleated arrays of fine actin filaments [161]. N-WASP – induced actin polymeration is also involved in neuronal growth factor (NGF)-induced differentiation of PC12 cells; intriguingly, some aspects of this process are inhibited by overexpression of the Exo70 subunit of the Exocyst complex, suggesting a strong link between the actin and membrane dynamics [162].

The Arp2/3 complex and its activators (SCAR/WAVE) play also a role in plant cell growth; although Arabidopsis loss-of-function mutants have mostly moderate phenotypic consequences affecting mainly trichomes, but also the shape and interdigitation of epidermal pavement cells (reviewed in [163]). Plant SCAR/WAVE- Arp2/3 module is activated by the Rop GEF SPIKE [164].

Major F-actin nucleators in land plants are, however, apparently the formins (FH2 proteins; [165-167]). Indeed, formins have entered the picture recently also in metazoan cells. The formins mDia1–3, cooperating with the Arp2/3 complex, are required for invadopodia assembly and function, [168], similar to the machinery acting in lamellipodia and filopodia [169]. In neurons, a different branch of the extensive formin family – the DAAM formins – participate in growth cone function [170].

Although the study of plant formins is hampered by functional overlaps within the large gene family, some observations indicate their participation in invasive tip growth. Heterologous expression or overexpresion of several plant formins caused loss of cell polarity in pollen tubes and/or changes in actin dynamics, mainly extensive bundling [144,171,172]. Overexpression of another Arabidopsis formin (AtFH8) elicited root hair depolarization and branching [173] while its non-functional derivative inhibited root hair growth [174].

Yeast and metazoan mDia-related formins are well-described direct effectors of RHO GTPases [175], whereas plant formins lack the characteristic conserved GTPase binding domain (FH3/GBD) and cannot thus be localized to the membrane by their interaction with Rho GTPases. Nevertheless, some plant formins contain a transmembrane domain (Class I) or a presumably lipid-binding PTEN domain (Class II; [165-167]). In addition, non-seed plants possess a third class of formins with a putative GTPase-binding domain related to RhoGAPs [167]. In invasive protonemata of the moss *Physcomitrella patens*, inhibition of PTEN-domain formins via RNAi results in isodiametric cell expansion [176].

While common mechanisms of actin nucleation involving the Arp2/3 complex and formins suggest a conserved molecular apparatus of cell invasion, lineage-specific pathways and “non-traditional” actin nucleators [177] may be contributing as well. In particular, a novel actin nucleator, Cordon-bleu (Cobl) negatively regulates neurite branching via promoting actin bundling [178].

### Myosins

Significant participation of the F-actin-associated motors, myosins, in the polarized/invasive cell growth was recently described in several models including root hairs and moss protonemal cells. It was found that elimination of the particular class XI myosins abolishes root hair growth or protonema elongation [179-183]. Furthermore, these myosins contribute to developmentally regulated organization of the F-actin bundles in the growing root hairs. In some combinatorial knockouts of myosin genes, the depolarization/branching defects (analogous to those seen when formin AtFH8 is up-regulated) were described [180]. Given that several plant myosins are expressed specifically in pollen [184], it seems likely that the myosins are indispensible for pollen tube growth as well. Although the roles of myosins in animal polar cell growth are less understood, myosin Va was found to pull ER intodendritic spines of the neurons [185]. Moreover, Myo10, an unconventional myosin with MyTH4-FERM domains, is required for the formation of invadopodia [26] and for the patterning of podosomes [186], invadopodia-like structures that are involved in integrin-dependent adhesion in cells such as osteoclasts.

To summarize, myosins emerge as important players in polar growth that contribute to the directed vesicle transport along the biosynthetic pathway [187,188] and to organization of the tip growth presumably via bundling or transporting F-actin [179,180].

### Additional players and actin-microtubule crosstalk

Additional actin-binding proteins contribute to the formation of invadopodia. Cortactin probably regulates the availability of cofilin to sever filaments to create new barbed ends for actin polymerization [189]. The actin-bundling protein fascin is critical for invadopodia stability [25]. Proteins that modify F-actin turnover participate also in the development of polarised neuronal protrusions. For instance, genetically modified mice lacking the F-actin severing protein gelsolin exhibit reduced migration of neuronal progenitors [190]. Many actin-binding and -regulating proteins known from animals and yeast have homologs also in tip growing plant cells (reviewed in [191]).

While microtubules are abundant in invadopodia [192], little is known about their specific roles in invasion. The microtubular cytoskeleton may participate mainly in later stages of invadopodia extension or subsequent cell migration rather than the early steps of substrate invasion *per se*[26,193].

Crosstalk between microfilaments and microtubules is apparently central to invasiveness of some cell types, including neurons (reviewed in [194-196]). Microtubules participate in extension of axonal branches, originally initiated mainly by actin-based mechanisms [197]. Axon-like neurites induced by cytochalasin D were enriched with dephosphorylated microtubule binding protein tau (Tau-1) and phosphorylated microtubule associated protein Map1b, known markers of polarisation [141]. Recent *in vivo* evidence from genetically modified mice suggests a specific role for the ubiquitously expressed plakin, microtubule-actin crosslinking factor 1 (MACF1), in migration of immature cortical pyramidal neurons [198,199]. Studies on non-neuronal cells suggest a possible mechanism, since MACF1 regulates the dynamics of F-actin-microtubule-focal adhesion interactions during keratinocyte migration [200]. In primary fibroblasts lacking MACF1, extending microtubules failed to co-align with F-actin filaments at the plasma membrane [201]. MACF1 enhanced the co-localisation of microfilaments and microtubules in transfected cells [199]. A human mutation in a neuro-specific tubulin isoform causes a complex phenotype whose underlying cause appears to be a defect in axon guidance [202].

In invasive plant cells, microtubules are less central than actin. Rather than growth itself, they appear to control its direction (e.g. [143,203,204]), and only extremely high concentrations of the microtubule-stabilizing drug taxol inhibited tobacco pollen tube elongation [205]. Sub-apical spiral bundles of cortical microtubules may serve as a “structural memory” enabling to restore growth direction after obstacle encounter [143,206]. Differential dependence of several pollen tube membrane proteins localization on actin and MTs cytoskeletons was recently described [207].

A family of plant-specific Ric (Rop interacting CRIB-domain) adaptor proteins was reported to mediate the communication between active Rop GTPases (see above) and both actin and microtubules [100,208]. These proteins also act in co-ordinating morphogenesis of neighbouring lobed epidermal pavement cells, enabling thus “mutual invasion” of adjacent cells resulting in a “puzzle-like” structure of the epidermis [209].

## Exocyst

### The exocyst is essential for cell invasiveness

Exocytosis directly contributes to invasive cell behaviour. Probably the best studied relevant effector of specific RHO (but also Rab) GTPases is the vesicle tethering complex exocyst – a hetero-octameric protein complex originally described as a Rab/Sec4 effector in yeast, consisting of Sec3, Sec5, Sec6, Sec8, Sec10, Sec15, Exo70 and Exo84 subunits, involved in the docking of exocytotic vesicles at the target cytoplasmic membrane before effective fusogenic t-v-SNARE complex formation [210,211]. While the exocyst is, in general, evolutionarily conserved, some subunits seem to be lost in some eukaryotic lineages [212], and the land-marking Exo70 subunits have extremely diversified in land plants [213-215].

In cancer cells the exocyst is essential for invadopodia formation and function and for invasiveness [84,216]. Knockdown of Exo70, Sec6, Sec8 or Sec10 decreased invadopodia number and/or reduced matrix degradation [84,216].

The exocyst is also essential for neuron polarisation and subsequent protrusive outgrowth. Expression of dominant negative mutants of Exo70 in cortical neuronal progenitors of mouse embryos disrupted glial-guided migration of pyramidal neurons [35]. Perturbation of exocyst function in cortical neurons *in vitro* reduced neurite branching and disrupted normal polarisation [217,218].

Plant exocyst is also implied in the invasive growth of pollen tubes and root hairs, as documented by phenotypes of *Arabidopsis* mutants [213,214,219,220].

### Mechanisms of exocyst function

The most obvious exocyst role in invasiveness is related to membrane trafficking, including, but not limited to, providing material for the expanding plasmalemma. In animal cells, exocyst participates also in the secretion of matrix-loosening enzymes [84,216]. In plants, a plethora of cell wall-regulating activities – expansins, extensins, cell wall polysaccharide-modifying enzymes – are exocytosed at the invading/growing cell domain. Mere relaxation of cell wall can induce polarized cell expansion due to manifestation of turgor pressure at the localized area of the cell surface [65,221]. Such local changes in cell wall properties usually tightly depend on localized exocytosis and membrane recycling.

Surprisingly, the exocyst also participates in cytoskeleton, especially actin remodelling, in particular via its association with the Arp2/3 actin nucleation complex. Mammalian Exo70 interacts with the Arpc1 subunit (Arc40 or p40) mediating the association of the whole exocyst to the Arp2/3 complex [222]. Exo70 and Arp2/3 complex co-localize at the leading edge of migrating cells, suggesting that Exo70 targets Arp2/3 to specific sites of plasma membrane [222]. Moreover, Exo70 stimulates Arp2/3 – mediated actin polymerization *in vitro*. Remarkably, the intensity of interaction between Exo70 and Arp2/3 correlated with invasive potential of cells, and expression of mutant Exo70 defective in interacting with Arp2/3 prevented invadopodia formation and matrix degradation [216]. Thus, association of Exo70 and Arp2/3 is essential for formation of invadopodia, and Exo70 may target Arp2/3 that subsequently mediates actin-based protrusions formation, required for cell migration.

Actin may not be the sole cytoskeletal efector of the exocyst, since both complete exocyst and its Exo70 subunits inhibit microtubule polymeriziation *in vitro* and Exo70 overexpression affects filopodia formation in rat kidney cells [223]. However, to date there are no microtubuli-related data directly related to cell invasiveness.

### Exocyst – small GTPase interactions

Regulatory links between the exocyst and small GTPases are well established in many systems. In invadopodia, the exocyst interacts with IQGAP1, a Cdc42 and Rac effector, which regulates cell polarization during cell migration [224] and was implicated also in tumorigenesis and invasion [225]. The interaction between IQGAP1 and Sec3/Sec8 is regulated by Cdc42 or RhoA, and is important for invadopodia formation [84]. Consistently, Exo70 and Sec8 are enriched in invadopodia, where they co-localize with F-actin, MMPs and IQGAP1. The exocyst in invadopodia apparently directs targeted MMP secretion; indeed, knockdown of IQGAP1 reduced MMP secretion similar to effects of exocyst inhibition [84,216]. Interaction of Exo70 with phosphatidylinositol-4,5-bisphosphate is necessary for MMP secretion [216].

No direct link between the exocyst and RHO GTPases was reported to date in neurons. However, the small GTPase Ral-A controls the ability of the neuronal exocyst proteins to associate with molecular regulators of polarity and protrusion [217]. Another small GTPase, TC10, interacts with Exo70 and triggers translocation of the exocyst to the plasma membrane during polarisation and neurite elongation; interestingly, the Exo70-TC101 complex can locally antagonize actin rearrangements induced by Cdc42 [162,226]. The absence of TC10 or Exo70 resulted in reduced elongation of neurites and the absence of axons, associated with the loss of polarised membrane insertion of the insulin growth factor -1 (IGF-1) receptor, a known regulator of axon specification [227].

In plants, the first link discovered between the exocyst and Rop GTPases is an indirect one through the Sec3 subunit interacting with a plant-specific adaptor protein Icr1, likely to participate in pollen germination and tube growth [228,229].

While there are no reports directly related to cell invasiveness, Rab GTPases are well-established exocyst effectors in both metazoans and fungi [230].

## Lipid signaling

### Invasive structures are membrane microdomains with specific lipid composition

Steroid- and sphingolipid-enriched membrane microdomains, often referred to as lipid rafts, play an important part in cellular invasive structures.

Invadopodia biogenesis and integrity rely on tightly controlled levels of plasma membrane cholesterol. Lipid raft perturbation e.g. by direct manipulation of cholesterol levels or by inhibition of glycosphingolipid synthesis impaired invadopodia formation and function [231,232]. Furthermore, caveolin 1 is a key regulator of plasma membrane cholesterol homeostasis required for invadopodia formation and ECM degradation, through a tyrosine-phosphorylation-dependent mechanism [231].

Similarly, a number of studies have shown the requirement for sphingolipid synthesis during outgrowth and morphological restructuring of neurites in cultured pyramidal neurons [233,234]. Interestingly, whereas during brain development cholesterol is autonomously generated by neurons, postnatally the majority is provided by supporting astrocytes, which release cholesterol-rich lipoproteins [235]. Thus, the role of astrocytes in supporting neurite outgrowth, synaptogenesis and regeneration in the brain is likely to be tightly linked with the regulated secretion of lipids [236].

In plants, sterols, which are characteristic for membrane lipid rafts, accumulate specifically at the point of bulging and at the tip during the active phase of Arabidopsis root hair elongation [237], and at growing pollen tube tips of spruce *Picea meyeri*, where their disruption by filipin inhibited growth [238]. Studies on Arabidopsis mutants defective in sterol biosythesis showed the crucial importance of sterol homeostasis for establishment and maintenance of plant cell polarity – especially for the endocytotic recycling-dependent dynamic polarization [239].

### Function of lipid rafts in invasive structures

In invadopodia, lipid rafts could participate in targeting signaling and proteolytic activities to degradation foci. Several invadopodia components can be regulated by, and preferentially sorted to, raft domains, including N-WASP, dynamin 2, MT1-MMP, integrins and ARF6 [240-244].

Lipid rafts also serve as scaffolds for membrane signalling molecules in neurons [245]. A correlation between cholesterol and the levels of raft-associated Fyn, a non-receptor tyrosine kinase known to affect neurite outgrowth, suggests a lipid-dependent signalling pathway controlling neurite growth [246,247]. Optimal levels of cholesterol may be essential for correct outgrowth of axons and dendrites in pyramidal neurons, since experimentally imposed changes in cholesterol levels attenuated outgrowth. Lower levels of cholesterol in cultured cortical neurons may account for faster neurite outgrowth when compared to hippocampal neurons [246]. Furthermore, exposure of neurons to cholesterol-reducing drugs, such as pravastatin, increased the rate of neurite outgrowth and branching through the inhibition of Rho isoprenylation and thus activity [248].

Many neural cell adhesion and transmembrane molecules involved in signal transduction during neurite outgrowth and pathfinding contain immunoglobulin-like (Ig) motifs and localise to lipid rafts [249,250]. Their recruitment to lipid rafts is dependent on the cytoskeleton, particularly F-actin [251]. The involvement of microtubules was demonstrated in cultured hippocampal neurons, where recombinant antibody-induced clustering of cholesterol and ganglioside GM1 on the cell surface promoted axon outgrowth via stabilisation of microtubules [252].

Membrane lipid raft fractions from plant cells are enriched in, among others, Rop GTPases, GPI-anchored proteins and NOX proteins [253,254], suggesting a role for lateral membrane compartmentation also in plant cell invasiveness. Indeed, sterol-rich microdomains were shown to be important for ROS-based signalling in *Picea meyeri* pollen tubes [238] Activated Rops were shown to be anchored into the detergent-resistant membrane fraction not only via prenylated C’-termini but additionally via reversible S-acylation at their N-termini [255].

In conclusion, the importance of sterol-rich membrane domains as sites where signalling cascades are originated to organise local actin remodelling [256,257] is very much in accord with the finding that invadopodia, but also neurites, plant root hairs and pollen tubes, are lipid-raft enriched domains.

### Phosphoinositide signaling and small GTPases at sites of invasion

An “Exocytic Signal” model suggesting that exocytosis and actin regulation are fully integrated events mediated by phosphoinositides (PtdIns)-based signaling has been recently proposed for polarized growth of fungal and metazoan cells [258]. PtdIns and their metabolism, which preferentially occurs at lipid rafts, participate also in invadopodia function, especially ECM degradation [259].

PtdIns(4,5)P_2_ is of special interest because it directly controls actin polymerization at the plasma membrane (reviewed in [260]). Both PtdIns(4,5)P_2_ and the kinase that generates it, type Iα PtdIns-4-phosphate 5-kinase, are enriched at invadopodia. Further, knockdown of the enzyme inhibited invadopodia formation and ECM degradation in a breast cancer cell line [261]. Class IA PtdIns3-kinase (PI3K), another lipid kinases that phosphorylate PtdIns, controls invadopodia formation in breast cancer cells, via its effectors 3-PtdIns–dependent protein kinase-1 (PDK1) and Akt [262]. Exocyst may be one of the relevant targets of PtdIns-based signalling, as suggested by the finding that PIP(2) binding to Exo70 participates in the control of cell motility, though it is not clear whether there is a direct role also in invasion into ECM [263].

Phosphoinositides are pivotal also for neurite formation and extension. In differentiating pyramidal neurons, activation of PI3K and generation of its product PtdIns(3,4,5)P_3_ occurs in a polarised manner and is associated with the specification and extension of the axon via the activation of Rap1B, Cdc42 and Rac1-dependent signalling [49]. Small GTPases provide a major signalling link between membrane lipids and the cytoskeleton in the developing forebrain [194]. Exposure of neurons to cholesterol-reducing drugs (see above) increased the rate of neurite outgrowth and branching through the inhibition of Rho isoprenylation and thus activity [248]. This is in accordance with the findings that in cultured neurons RhoA can have oposite biological effects from Rac1 and Cdc42, inhibiting rather than promoting neurite outgrowth [264]. Furthermore, phospholipase C 3 (PLC 3), a key neuronal enzyme catalyzing the hydrolysis of PI(4,5)P2 and leading to the generation of second messengers diacylglycerol (DAG) and inositol 1,4,5-trisphosphate (IP3), promotes neurite outgrowth through downregulation of RhoA [265].

PtdIns participate in invasive growth also in plant cells, since the first characterized plant membrane phospholipid-regulating activity (PtdInsP-kinase) was reported as an effector of active Rop at the pollen tube tips [266]. Rapidly growing evidence indicates an important role for both PI3K and PI(4,5)P2 in pollen tube growth [267-269]. Specific Rab GTPases of the Rab11/A clade and all Rab8/E paralogues are currently the best known regulators of PtdInsP-kinases in Arabidopsis root hairs [270,271]. These activities contribute to the establishment and function of the highly dynamic PIP2-rich plasmalemma domain at the growing tips of pollen tubes and root hairs (reviewed in [113]). Phospholipase D (PLD) and its product - phosphatidic acid (PA) is emerging as a regulator able to link F-actin cytoskeleton and membrane lipid dynamics in plants. While in animal cells F-actin polymerization is stimulated by binding of capping protein to PIP2, plant actin capping protein is inhibited by PA. At the same time in both cell types F-actin directly stimulates PLD activity, resulting in plant cells in positive feedback, locally amplifying both membrane and actin dynamics (reviewed in [272]).

## Reactive oxygen species (ROS)

### ROS as second messengers contributing to cell invasiveness

Reactive oxygen species (ROS), while best known for their function in pathological processes, are also essential intracellular second messengers in many signaling pathways, including those related to cell invasiveness. In cells, ROS are produced by several mechanisms, including the activity of NADPH oxidases (Nox), transmembrane proteins that catalyze NADPH-dependent formation of superoxide from oxygen via an electrogenic charge/electron transport (reviewed in [273]).

Both ROS and Nox proteins are essential for formation and function of invadopodia, inducing ECM degradation and cell invasiveness. Because ROS are very unstable, they must be generated locally. ROS production is targeted to invadopodia by Nox organizer proteins Tks4 (tyrosine kinase substrate with 4 SH3 domains) and Tks5 (tyrosine kinase substrate with 5 SH3 domains), two large scaffold proteins targeted to invadopodia by interaction with PI-3,4-P_2_. Tks proteins interact both with Nox activator proteins and Nox core enzymes, thereby inducing formation of an active Nox complex [274,275]. Further mechanisms whereby ROS regulate invadopodia formation and function are not yet elucidated.

ROS are also required during neural development and function, including the proliferation of neuronal precursors, their differentiation into specific neuronal subtypes, as well as the survival and the plasticity of adult neurons. Cultured rat cortical neurons, as well as CNS areas rich in immature, migrating neurons, have high levels of ROS [276,277].

Changes in ROS levels influenced the types of neurons produced *in vitro*, suggesting levels of ROS in neuronal progenitors control neuronal fate decisions. Intracellular synthesis of ROS in neuronal progenitors may be regulated by the cell fate determining factor Numb and its associated protein Nip1/Duoxa1 (Mammalian Numb-interacting Protein 1/Dual Oxidase Maturation Factor 1)[278]. ROS have been also shown to be essential for the NGF-induced differentiation of neuron-like, rat pheochromocytoma PC12 cells [279-281] via its receptor TrkA [282].

In tip-growing plant cells, polarized production of ROS depends on a high tip-focused free calcium ion (Ca2+) cytoplasmic gradient correlated with invasive growth of pollen tubes [283,284]. The gradient is regulated by glutamate receptor-like calcium channels activated by D-Ser in transmitting tract of pistils [285]. A similar tip-focused Ca2+ gradient was described also in root hairs [70,286,287]. The cytoplasmic free calcium gradient connected to apical invasive growth seems to be regulated by active Rop GTPases via Ric proteins [100]. First indirect interaction of Rop GTPase, mediated via an Icr-like/Rip3 adaptor protein binding a specific kinesin, was reported recently by Mucha et al. [288]; for Icr1 see [228]). ROS-producing NADPH oxidases (Nox) are prominent candidate integral membrane proteins regulated directly by calcium and activated Rho GTPases also in plants, as demonstrated initially by forward genetic screens in Arabidopsis root hairs [103,289] and later by pharmacological and antisense supression of NOX activity also in pollen tubes [290].

### ROS signaling networks in invasive structures

The mechanisms of ROS effects in invadopodia formation and function are not well understood yet. ROS can oxidize redox-sensitive cysteins in catalytic sites of many proteins, including protein tyrosine phosphatases, generating thus sites with enhanced tyrosine kinase activity. Particularly, ROS can inactivate protein tyrosine phosphatase PTP-PEST that localize to invadopodia or LMW-PTP, resulting in enhancing protein tyrosine kinase activity, especially of Src kinase, that was shown to be essential for invadopodia function. Src kinase can act both upstream and downstream of this signaling. On one hand, ROS production enhances Src activity, on the other hand, Src phosphorylates Tks and NOX proteins to strengthen their interaction and promote enhanced Nox activity. Another mechanism of ROS effect in invadopodia can be regulation of MMPs secretion by fusion of MMPs-containing vesicles with plasma membrane (reviewed in [291,292]).

The association of ROS signaling and RHO GTPases was described in both non-neuronal cells and in neurons. In invadopodia, Src can induce ROS generation by activating Rac1 GEF Vav2. Rac1 in turn activates Nox complexes to produce ROS [293]. In *Aplysia* neurons, ROS are likely to promote neurite outgrowth via inhibition of Rho. Interestingly, in PC12 cells Rac1 can also increase intracellular ROS levels by activating Nox complexes [294] or the cytosolic phospholipase A2/arachidonic acid/lipoxygenase-cascade [295]. Interference in Rac signalling by over-expression of a catalytically inactive mutant, RacN17, blocked the NGF-induced generation of ROS and morphological differentiation of PC12 cells [281]. STAT3 was also found to control NGF-induced ROS production during process outgrowth in PC12 cells suggesting broad involvement of ROS in multiple signaling pathways [296].

While plant NOX proteins are directly regulated by calcium, phosphorylation [290,297,298] and by binding to active Rop GTPases, positive regulatory feedback between NOX-ROS and ROS-activated calcium channels is proposed to operate at the growing tip contributing to the tip-high cytoplasmic free calcium gradient both in pollen tubes and root hairs [290,298].

ROS could also have a direct impact on the cytoskeleton. Localized synthesis of ROS in *Aplysia* growth cones is required for F-actin assembly and dynamics. Short term exposure to a free radical scavenger, or inhibition of ROS sources such as NADPH oxidases and lipoxygenases, reduced the F-actin content in the peripheral domain of growth cones [299]. Further, prolonged treatment resulted in the disassembly of the actin cytoskeleton, causing severely impaired growth cone formation and outgrowth.

In plant cellsROS-mediated cell wall polysaccharide cleavage/relaxation or cross-linking may contribute to the regulation of cell expansion (reviewed in [300]). NOX-ROS might also modify local membrane lipid composition by lipid peroxidation and play important roles in membrane lipid raft organization.

## Review and conclusions

### Is there an ancestral toolbox for cell invasiveness?

Comparison of cell invasiveness mechanisms in lineages as diverse as plants and metazoans should provide hints towards reconstruction of a potential set of ancestral mechanisms and pathways that may have been at the root of the phenomenon of eukaryotic cell invasiveness. Indeed, many structural and signalling motifs appear to be common or conserved (see Table 1). The closely interconnected membrane trafficking processes and actin filament rearrangements, orchestrated by small GTPases and integrating multiple signalling inputs, in particular those utilizing (phospho)lipid-protein interactions and reactive oxygen species, appear to be a recurrent theme in evolutionally distant lineages and thus good candidates for possible ancestral mechanisms, possibly reaching back to the LECA. Regardless of what LECA looked like, it likely have had large cells and lived in an anisotropic environment, necessitating cellular polarity to navigate in gradients of physical and chemical inputs.. Invasivness of present eukaryotic cells then appears as a natural consequence of growth or locomotion of their polarized ancestors in semi-solid substrates such as e.g. sediments.

**Table 1:** Comparison of cell invasivity mechanisms in model cell types

**Process or component**	**Invadopodia**	**Neurites**	**Pollen tubes and root hairs**
**Matrix loosening or detachment**	Yes (proteases)	Yes (SPARC-like1, reelin)	Yes (xylanases in pollen tubes, secretion of mucilage facilitating movement through soil in root hairs)
**Branched actin network at or close to leading edge or tip**	Yes (structural role)	Yes	Yes (delivery of secretory vesicles)
**Actin nucleation mechanism(s) involved**	Arp2/3, formins (mDia1-3)	Arp2/3, formins (DAAM)	Formins
**Microtubules required**	Yes (for extension)	Partly (for guidance or orientation)	Partly (for orientation)
**RHO class GTPases involved**	Cdc42	Rac1, Cdc42, RhoA (predominantly Rac1)	Rop
**ARF class GTPases involved**	Arf6	Arf6	Arf1
**RAB class GTPases involved**	Rab8, Rab25, Rab27	Rab8, Rab17, Rab22	Rab8/RabE, Rab11/RabA, Rab1/RabD
**Exocyst**	Yes (via Cdc42, RhoA and Arp2/3; for MMPs secretion and actin polymerization)	Yes (via RalA and TC10)	Yes (via Rop – maybe indirectly – and Rab; for membrane turnover and cell wall modifications)
**Membrane microdomains as scaffolds for signalling**	Cholesterol, sphingolipids	Cholesterol, sphingolipids	Sterols
**PtdIns signalling**	Yes (PtdIns(4,5)P_2_)	Yes (PtdIns(3,4,5)P_3_, DAG, IP3)	Yes (PtdIns(4,5)P_2_, PtdIns(3,4,5)P_3_)
**ROS signalling**	Localized production by Nox regulated via Src	Via Nip1/Duoxa1 or NGF, role in differentiation	Localized production by Nox regulated via Ca^2+^

**Figure 2:**
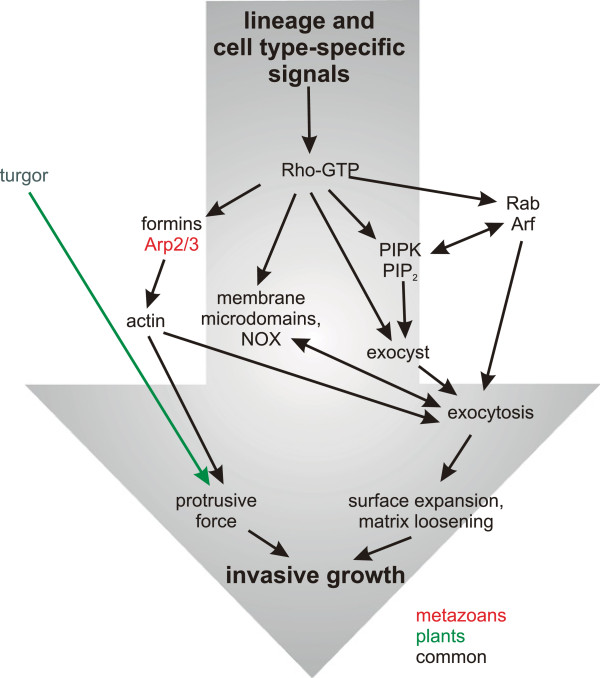
**Summary of conserved regulatory and functional pathways responsible for invasive growth in eukaryotic cells discussed in this review. **Components shared by plants and metazoans are shown in black, metazoan- and plant-specific ones in red and green, respectively.

However, while homology of individual proteins can be readily assessed, the situation is much less clear in case of cellular processes where conserved components could have combined in varying manner to achieve convergent overall network topology (see Figure 2). Nevertheless, the Rho/Rac/Rop GTPases are apparently as old as the eukaryotes [301], and they a playing a comparable part in organisms as diverse as opisthokonts and plants. Thus they emerge as good candidates for a group of truly conserved ancestral master polarity regulators. While we cannot exclude the possibility that some of the components of the pathways leading from the small GTPases to their ultimate cytoskeletal or secretory effectors may have been recruited from a common cellular toolbox independently, i.e. in a convergent fashion, it is plausible that even these mechanisms are, at least to some extent, ancestral. This, however, does not exclude lineage-specific “implementation” of some regulatory steps, as can be illustrated e.g.in case of RHO-dependent control of actin nucleation [302]. Both small G proteins and actin do have distinguishable prokaryotic homologs. Albeit the relationship of the bacterial GTPases to distinct eukaryotic clades remains unresolved [301,303], at least in some case a bacterial small GTPase was found to contribute to cell polarity control [304], opening up the intriguing possibility that some of the machinery involved in invasiveness might have predated LECA,

Striking similarity between cell invasiveness mechanisms in plants and metazoans, including pathological conditions such as invasive cancer, opens great possibilities for transfer of findings related to invasive cell growth from one biological system into the others. As an example, the role of exocyst and NADPH oxidases in invasive pollen tube growth was discovered earlier than the role of these signalling modules for cancer cell invasion. Such a transfer of findings, although cautious, could result in facilitation of the research focused on treatment of pathological conditions related to invasive cell growth, including, e.g., also the metastatic cancer and neuronal regeneration.

## Reviewers comments

### Reviewer's report 1: Arcady Mushegian, Kansas University Medical Center, United States of America

The manuscript by Vaškovi#ová et al., poses an interesting evolutionary and functional question, namely whether the mechanisms of cell invasive growth in plants and animals share homologous molecular components. The study is the review of the evidence, which gives an affirmative answer: the examples of cell invasion that are selected for the review are all dependent on the rearrangements of actin cytoskeleton, controlled by small GTPases of Rho, ARF and Rab families. Moreover, in both plants and animals the lipid (phosphoinositol) signalling is involved, as well as possibly membrane microdomains.

I have no objection against publication of this review as is, but the authors are invited to consider whether some of the following modifications would improve the manuscript.

First, the definition of invasive growth is, in my opinion, loose. Invasion surely implies growing through, or within, another living tissue; the authors suggest that much, pointing out the importance of the digestion of the intercellular matrix, for which the appropriate enzymes should be secreted. If this is a necessary part of the definition, then pollen tube growth qualifies, but the growth of root hairs seems not to: secretion of “lubricants” is surely not the same as secretion of active lytic enzymes, or is it - lubricants do not have even to be proteins? On the other hand, growth of haustoria of parasitic plants seem to qualify in every way.

Author response: *We realize that we should have taken more care to define invasiveness at the first place, and we have now corrected this omission. In our opinion (and in agreement with Reviewer 2), the environment invaded by the cells does not need to be a living tissue - it is sufficient that it is semi-solid. In real-life situations, the environments are also usually rather complex (soil is a good example of this).*

Secretion of both the digestive enzymes and other materials such as the root hair mucilage (i.e. polysaccharides, glycoproteins) or even small molecules critically depends on a local activity of the exocytotic pathway, and at least in this sense the inclusion of root hairs can be justified. From this point of view, we would not consider the haustoria a relevant model for cellular invasiveness, as they are multicellular structures.

Second, the (near) absence of fungi in the discussion is puzzling. Surely, if root hairs qualify, fungal hyphae should too; but even if not, the growth of parasitic fungi is invasive, and, at least in the case of Candida and some plant parasites even reasonably well studied genetically. In fact, the names of many genes involved in the process, notably Cdc42, are from the fungal systems!

Author response: *We agree that fungi, in particular the budding yeast, have served as paradigmatic models for the study of cell polarity and cell invasion. We have now added an explanation of the reasoning behind our choice of models, as well as references to recent reviews on cell polarity and hyphal growth in fungi.*

Third, if at least some components of the invadopodia are shared between plants and animals - and also fungi, if the authors will be willing to talk about them, too - then an inference is that these components were also present in LECA. Were they organized into a coherent system, and what that system might have been?

A putative answer presents itself, i.e., these components, including cortical/apical actin cytoskeleton, GTP regulators, and associated membrane remodeling components may have been involved in forming pseudopodia and other cytoplasmic protrusions. In fact, among such protrusions in any organisms, the best-studied one from genetic point of view may be S.cerevisiae bud, which is very much controlled by Cdc42 and other players discussed in the review. It seems to be worth discussing the present-day unicellular eukaryotes more explicitly, even though they are obviously not LECA.

Author response: *We have also now expanded somewhat the concluding section on the “ancestral toolbox of cell invasiveness”, suggesting that the RHO clade GTPases emerge as possible candidates for truly conserved invasivity regulators. While we do not dare to postulate that LECA was capable of forming pseudopodia or similar protrusions, we do believe that it was polar, and that this alone may have provided a sufficient starting point for the evolution of invasiveness.*

### Reviewer’s report 2: Valerian Dolja, Oregon State University, United States of America

This review article discusses parallels and differences in the molecular mechanisms of the polarized cell elongation in animals and pants interpreted as cell invasiveness (I think that invasiveness is a better term than ‘inasivity’ which does not appear in Webster). Both the cell growth within the tissue (as in neurons, cancer cells, or pollen tubes), and into the surrounding substrate (as in root hairs) are considered under the common umbrella, and rightfully so. The mechanisms of invasive growth are interpreted in evolutionary terms; a much needed, rather refreshing, and enjoyable prospective in the field of cell biology. I found the article very engaging, well written and useful for the broad audience interested in the interplay of vesicle transport, cytoskeleton, lipid signaling, and ROS.

Author response: *Thanks for suggesting the term “invasiveness” - we are now using it throughout.*

There are two major comments I would like the authors to consider. The first has to do with relating commonalities/homologies in the tip growth mechanisms in animals and plants to LECA. The supergroups of plants and unikonts are indeed separated from each other as deeply as it goes among the eukaryotes, likely all the way to LECA. However, it does not mean that the plant and animal homologous proteins involved in invasive growth (e.g., small GTPases) have evolved to fulfill this role. Because LECA was certainly unicellular, it seems more likely that the small GTPases, and other components of polar growth machinery were recruited from available toolbox later, along with evolving multicellularity and tissue differentiation.

Author response: *Although we agree that LECA lacked the features we associate with advanced multicellularity (i.e. cell diferentiation), we are not that sure that it was unicellular, as it may have lived in some form of colonies or consortia. Even modern day prokaryotes can form rather sophisticated multicellular structures, justifying even the application of the concept of a “body plan” (see Rieger et al., Commun Integr Biol. 2008 1(1): 78–8). However, we do not consider multicellularity essential for invasiveness - a need to navigate in a complex semi-solid environment may have been enough, and the heterogeneity of the environment naturally resulted in cell polarity (be it even “only” due to the gradient of environmental conditions). Small GTPases may have been recruited as “master regulators” of diverse pathways related to cell polarity already at this stage, while subsequent - more diverse - steps of the pathways may have been either also inherited or independently recruited. We are now discussing this hypothesis in the final section.*

The second comment has to do with the missing discussion of significant roles played by the F-actin-associated motors, myosins, in the polarized/invasive cell growth. These roles were recently described in several models including root hairs and moss protonemal cells. It was found that elimination of the particular class XI myosins abolishes root hair growth or protonema elongation [1-5]. Furthermore, these myosins contribute to developmentally regulated organization of the F-actin bundles in the growing root hairs; in some multiple knockouts of myosin genes, the depolarization/branching defects (analogous to those seen when AtFH8 is up-regulated) were described [4]. Given that several plant myosins are expressed specifically in pollen [6], it seems likely that the myosins are indispensible for pollen tube growth as well. Although polar growth of the budding yeast cells cannot be considered invasive, it also relies on myosin V closely related to plant myosins XI [7]. Although the roles of myosins V in animal polar cell growth are less understood, myosin Va was found to pull ER into dendritic spines of the neurons [8]. Therefore, myosins emerge as important players in polar growth that contribute to the directed vesicle transport along the biosynthetic pathway [9,10] and to organization of the tip growth area presumably via bundling or transporting F-actin [4,5].

Author response: *We thank the reviewer for pointing out the missing discussion on the role of myosins in invasive growth. We now included a subchapter “Myosins” in Cytoskeleton(s) part of our review, where the role of myosins is discussed.*

### Reviewer’s report 3: Purificacion Lopez-Garcia, Centre National de la Recherche Scientifique, France

This is an interesting and synthetic review on the molecular mechanisms and effectors responsible for cell invasive growth in animals and plants, where most data are available. The review highlights the similarities and homologies existing between both so that, at the end, the question of whether such mechanisms existed already in the last common eukaryotic ancestor (LECA) can be asked.

I have few comments on the review part, which is very well documented and naturally oriented to the final question asked in the manuscript. Perhaps, it would have been important to mention data existing for other eukaryotic phyla whenever available. Animals and plants are divergent in the eukaryotic tree, but the possibility that they have shared a most recent common ancestor (for instance, to the exclusion of excavates) cannot be completely ruled out.

Author response: *With the exception of fungi, which are, however, rather close to metazoans, surprisingly little is known about mechanisms of invasiveness in other major phyla (we did include this comment and a reference to recent - mostly cytological - Phytophtora work in the new version of the paper).*

At any rate, the question of whether invasive cell growth mechanisms and effectors existed already in LECA is posed in a very cautious and reasoned (even shy) manner. The authors highlight the fact that even if individual proteins are homologous in animals and plants and, therefore, potentially ancestral, the processes themselves may have resulted from the combination of different effectors in the two eukaryotic lineages. Although not clearly stated, the authors seem to suggest by this that the processes for invasive growth were present in LECA but that different combinations of effectors occurred in different eukaryotic lineages. This would imply a last common ancestry for both, effectors and processes. However, it might also be that from a same pool of ancestral proteins, the same or different proteins were recruited independently for a similar function, invasive growth, in later eukaryotic evolution, i.e. evolved by convergence from a common pool of proteins. The two possibilities should be perhaps more clearly distinguished.

Author response: *Since the question of distinguishing conserved invasiveness mechanisms from those recruited convergently from a common “toolbox” has been raised by all three reviewers, we attempted to respond to it by expanding the final discussion.*

Finally, the authors did not evoke the logical next question: do these proteinsn involved in invasive growth have homologs in prokaryotes? Do they play a role in analogous processes (e.g. cell polarization and gliding in social bacteria)? Describing those processes in bacteria is likely out the scope of this manuscript, but a comment on these might underscore the evolutionary orientation of this review.

Author response: *We do agree that prokaryotes present a fascinating area of research that would be well worth a separate paper; we did, at least, include a small note on possible prokaryotic homologs of some of the components in the expanded final discussion.*

Please, do not use “kingdom” to refer to plants, animals and fungi. The 5-kingdom classification by Witthaker has no phylogenetic support.

Author response: *While we did not use the term “kingdom” in the Whittaker sense, but rather in a looser sense of a “lineage”, we agree that it is better to get rid of this term completely, which we did.*

## Abbreviations

ARF: ADP-ribosylation factor; Cobl: Cordon-bleu; DAG: Diacylglycerol; ECM: Extracellular matrix; GAPs: GTPase-activating proteins; GDI: GDP dissociation inhibitor; GDP/GTP: Exchange factor; GDP: Guanosine diphosphate; GTP: Guanosine triphosphate; Ig: Immunoglobulin-like; IGF-1: Insulin growth factor -1; IP3: 1,4,5-trisphosphate; LECA: Last common eukaryotic ancestor; MACF1: Microtubule-actin crosslinking factor 1; NGF: Neuronal growth factor; Nox: NADPH oxidases; PA: Phosphatidic acid; Pak-1: p21-activated kinase; PDK1: 3-PtdIns–dependent protein kinase-1; PI3K: PtdIns3-kinase; PLCd3: Phospholipase Cd3; PLD: Phospholipase D; PtdIns: Phosphoinositides; ROS: Reactive oxygen species; SPARC: Secreted protein acidic and rich in cysteine; TGN: Trans golgi network.

## Competing interests

The authors declare that they have no competing interests.

## Authors’ contributions

All authors participated in writing and editing various portions of the manuscript. The final editing was done by KV, JB, DR and FC. All authors read and approved the final manuscript.
